# Distinct effects of ubiquitin overexpression on NMJ structure and motor performance in mice expressing catalytically inactive USP14

**DOI:** 10.3389/fnmol.2015.00011

**Published:** 2015-04-23

**Authors:** Jada H. Vaden, Jennifer A. Watson, Alan D. Howard, Ping-Chung Chen, Julie A. Wilson, Scott M. Wilson

**Affiliations:** ^1^Evelyn F. McKnight Brain Institute, Department of Neurobiology and Civitan International Research Center, University of Alabama at BirminghamBirmingham, AL, USA; ^2^Department of Structural Biology, St. Jude Children’s Research HospitalMemphis, TN, USA

**Keywords:** neuromuscular junction, ubiquitin, USP14, pJNK, proteasomes, motor neuron and deubiquitinating enzyme

## Abstract

Ubiquitin-specific protease 14 (USP14) is a major deubiquitinating enzyme and a key determinant of neuromuscular junction (NMJ) structure and function. We have previously reported dramatic ubiquitin depletion in the nervous systems of the USP14-deficient *ataxia* (*ax^*J*^)* mice and demonstrated that transgenic ubiquitin overexpression partially rescues the *ax^*J*^* neuromuscular phenotype. However, later work has shown that ubiquitin overexpression does not correct the *ax^*J*^* deficits in hippocampal short term plasticity, and that transgenic expression of a catalytically inactive form of USP14 in the nervous system mimics the neuromuscular phenotype observed in the *ax^*J*^* mice, but causes a only a modest reduction of free ubiquitin. Instead, increased ubiquitin conjugates and aberrant activation of pJNK are observed in the nervous systems of the USP14 catalytic mutant mice. In this report, we demonstrate that restoring free ubiquitin levels in the USP14 catalytic mutant mice improved NMJ structure and reduced pJNK accumulation in motor neuron terminals, but had a negative impact on measures of NMJ function, such as motor performance and muscle development. Transgenic expression of ubiquitin had a dose-dependent effect on NMJ function in wild type mice: moderate levels of overexpression improved NMJ function while more robust ubiquitin overexpression reduced muscle development and motor coordination. Combined, these results suggest that maintenance of free ubiquitin levels by USP14 contributes to NMJ structure, but that USP14 regulates NMJ function through a separate pathway.

## Introduction

Ubiquitination can regulate a diverse array of cellular pathways depending on the number of ubiquitin monomers conjugated onto a protein and the internal lysine residue used to form the ubiquitin chain. These pathways regulate protein stability (Hershko and Ciechanover, 1998), activity (Schmukle and Walczak, 2012; Humphrey et al., 2013; Zhou et al., 2013), and localization (Tanno and Komada, 2013). The accumulation of ubiquitin-positive aggregates is a hallmark of neurodegenerative diseases (Perry et al., 1987; Lowe et al., 1988; Jara et al., 2013), suggesting that sequestration of ubiquitin, and the consequent reduction of ubiquitin availability, could contribute to neuronal dysfunction. This suggestion is supported by the hypothalamic dysfunction and neurodegeneration observed in mice lacking the ubiquitin gene *Ubb* (Ryu et al., 2008).

Although ubiquitin is encoded by four genes in the mammalian genome (Lund et al., 1985; Wiborg et al., 1985; Baker and Board, 1987, 1991; Finley et al., 1989; Redman and Rechsteiner, 1989), the equilibrium between ubiquitin conjugated onto proteins and free ubiquitin is largely controlled by the opposing actions of ubiquitin ligases and deubiquitinating enzymes (DUBs; Hallengren et al., 2013). We have previously shown that loss of the proteasome-associated DUB ubiquitin-specific protease 14 (USP14) in the *ataxia* (*ax^J^*) mice results in perinatal lethality, reduced muscle development, and structural and functional defects at the neuromuscular junction (NMJ). Loss of USP14 also severely alters ubiquitin homeostasis by causing a dramatic depletion of free ubiquitin in the brain and spinal cord (Anderson et al., 2005; Chen et al., 2009). Ubiquitin levels were most severely affected at the synapse, suggesting that the NMJ deficits in the *ax^J^* mice are due to ubiquitin depletion (Chen et al., 2009). In fact, neuronal-specific transgenic expression of ubiquitin corrects the reduced muscle development, altered NMJ structure, reduced synaptic transmission, and perinatal lethality observed in the *ax^J^* mice (Chen et al., 2011). Although these findings suggest a role for USP14’s in the maintenance of free ubiquitin pools in the nervous system, later work showed ubiquitin complementation does not rescue the deficits in hippocampal short-term plasticity observed in the *ax^J^* mice (Walters et al., 2014).

Furthermore, we recently reported that transgenic expression of a dominant-negative, catalytically inactive USP14 species in the murine nervous system recreates many of the essential phenotypes observed in the *ax^J^* mice, including deficits in NMJ structure, poor motor performance, and reduced muscle development (Vaden et al., 2015). In contrast to the *ax^J^* mice, however, the USP14 catalytic mutant mice have a normal lifespan and only a modest decrease in free ubiquitin. Instead, the spinal cords of the USP14 catalytic mutant mice show an increase in levels of ubiquitin conjugates linked through lysine 63 (K63), which are known to promote kinase activation (Yang et al., 2010; Humphrey et al., 2013). Consistent with this finding, the USP14 catalytic mutant mice have increased activation of the ubiquitin-dependent mixed lineage kinase 3 (MLK3) pathway, which signals through c-Jun N-terminal kinase (JNK; Vaden et al., 2015). As expected from the well-documented role of pJNK in rearranging synaptic architecture in invertebrates (Etter et al., 2005; Collins et al., 2006; Drerup and Nechiporuk, 2013), *in vivo* inhibition of JNK significantly improved NMJ structure and muscle development in the USP14 catalytic mutant mice (Vaden et al., 2015).

These findings led us to hypothesize that, in addition to maintaining ubiquitin homeostasis, USP14’s catalytic activity is required for the termination of ubiquitin signaling cascades and, consequently, that the increase in ubiquitin conjugates observed in the spinal cords of the USP14 catalytic mutant mice can alter neuronal function independent of the reduction of free ubiquitin. To directly test this hypothesis, we generated mice expressing both catalytically inactive USP14 (Tg*CA*) and ubiquitin (Tg*Ub*) under the neuronal *Thy1.2* promoter. The spinal cords of the resulting Tg*CA*, Tg*Ub* mice had control levels of free ubiquitin and a nearly twofold increase in ubiquitin conjugates. The motor performance and muscle development of the double transgenic Tg*CA*,Tg*Ub* mice were reduced compared to both Tg*CA* mice and controls, underlining the need for USP14-dependent deubiquitination events in the nervous system. However, despite the detrimental effects of ubiquitin complementation on the overall phenotype of the Tg*CA* mice, the structure of the NMJ was improved, and the amount of pJNK-positive pathology was decreased, in the Tg*CA*,Tg*Ub* mice compared to Tg*CA* mice. Additionally, we found that overexpression of ubiquitin had a bidirectional, dose-dependent effect on muscle mass, motor function, and measures of synaptic transmission even in wild type mice where USP14’s activity was intact. These data suggest that ubiquitin complementation in *ax^J^* mice indirectly corrects the functional deficits caused by loss of USP14 and demonstrate that increased protein ubiquitination alters motor function.

## Materials and Methods

### Animals

Wild type C57BL/6J, *Thy1-YFP* mice (16JRS, Jackson Laboratories, Bar Harbor, ME, USA), transgenic mice expressing USP14 (Tg*Usp14)* catalytically inactive USP14 (Tg*CA;* previously referred to as Tg*Usp14CA*), ubiquitin (Tg*Ub*), or both catalytically inactive USP14 and ubiquitin (Tg*CA*,Tg*Ub*) have been maintained in our breeding colony at the University of Alabama at Birmingham, which is fully accredited by the Association for Assessment and Accreditation of Laboratory Animal Care International. We have previously described the generation of the Tg*Usp14* (Crimmins et al., 2006), and Tg*Ub* (Chen et al., 2011), and Tg*CA* mice (Vaden et al., 2015), and all transgenes are expressed from the neuronal *Thy1.2* promoter. Generation of Tg*Ub* mice resulted in two different founder lines with differing ubiquitin expression, Tg*Ub-H* (high expresser) and Tg*Ub* (low expresser). Double transgenic (Tg*CA*,Tg*Ub*) mice were generated by breeding male Tg*CA* mice to female Tg*Ub* mice. All mouse lines were maintained on a C57BL/6J background and transgenic mice were heterozygous for the transgene(s) of interest. Research was conducted without bias toward the sex of animals used for each study, and equal numbers of male and female mice were used. All research complied with the United States Animal Welfare Act and other federal statutes and regulations relating to animals and experiments involving animals, and adhered to principles stated in the Guide for the Care and Use of Laboratory Animals, United States National Research Council. In addition, all experiments were carried out with the approval of the University of Alabama at Birmingham’s Institutional Animal Care and Use Committee.

### Isolation of Proteins

Mice were deeply anesthetized with isoflurane prior to rapid decapitation. Spinal cords were removed and homogenized in modified RIPA buffer containing 50 mM Tris, pH 7.5, 150 mM NaCl, 5 mM MgCl_2_, 0.5 mM EGTA, 1 mM EDTA, 0.5% SDS, 1% Triton X-100, and 1% sodium deoxycholate. Complete protease inhibitor (Roche, Indianapolis, IN, USA), phosphatase inhibitor cocktail III (Sigma Aldrich, St. Louis, MO, USA), and 50 μM PR-619 (inhibitor of DUBs, Life Sensors, Malvern, PA, USA) were added to the homogenization buffer per manufacturer instructions. Following homogenization, samples were sonicated and centrifuged at 17,000 × *g* for 10 min at 4°C. The supernatants were removed and stored immediately at -80°C. Protein concentrations were determined using the bicinchoninic acid (BCA) protein assay kit from Pierce (Rockford, IL, USA).

### Immunoblotting and Quantitation

Proteins were resolved on 10% Tris-glycine gels and transferred onto nitrocellulose membranes. BSA (2%) in PBS containing 0.1% NP-40 was used to block the membranes and dilute the primary and secondary antibodies. HRP-conjugated secondary antibodies (Southern Biotechnology Associates, Birmingham, AL, USA) and SuperSignal West Pico Chemiluminescent Substrate (Thermo Scientific, Rockford, IL, USA) were used for detection. For detection of ubiquitin, proteins were resolved on 4–12% NuPage Tris-bis gels (Life Technologies, Grand Island, NY, USA) and transferred onto PVDF membranes. Membranes were treated with 0.1% glutaraldehyde in PBS for 20 min prior to blocking in PBS containing 2% BSA and 0.1% NP-40. The secondary antibody was diluted into PBS containing 1% non-fat dry milk and 0.1% NP-40. For quantitation, all blots were scanned using a Hewlett-Packard Scanjet 3970, and band density was quantified with UN-SCAN-IT gel digitizing software (Silk Scientific, Inc., Orem, UT, USA).

### Antibodies

The following antibodies were used: USP14 (Anderson et al., 2005), β-tubulin (Developmental Studies Hybridoma Bank, Iowa City, IA, USA); Ubiquitin (UAB Hybridoma Facility, Birmingham, AL, USA); pJNK, and JNK (Cell Signaling Technology, Danvers, MA, USA).

### Open Field

Animals were handled 1 day prior to open field testing. Locomotor activity was measured in an open field chamber (43.2 cm × 43.2 cm × 30.5 cm) for 15 min by an automated video tracking system (Med Associated, St. Albans, VT, USA). The first 5 min were not analyzed to account for habituation to the chamber.

### Rotarod

Motor coordination was tested by placing mice on a rotating rod (ENV-575, Med Associates), which accelerated from 3.5 to 35 rpm over a 5-min period. Latency to fall was recorded over three trials, each separated by 1 h, and the individual trials for each animal were averaged.

### Quantitative PCR

Total RNA was isolated from gastrocnemius muscles or spinal cords using RNA-STAT60 (Tel-Test, Friendswood, TX, USA) and reverse transcribed using the Superscript VILO cDNA synthesis kit (Life Technologies) per manufacturer instructions. Individual gene assays were purchased from Applied Biosystems for each of the RNAs analyzed: *AChR*-α (Mm00431629_m1), *AChR-*ε (Mm00437411_m1), and *AChR-*γ (Mm00437419_m1). ΔΔCt values were generated using *Gapdh* (Mm99999915_g1) as an internal standard. All values are reported as mean ± SEM of at least three different animals per genotype, run in triplicate.

### NMJ Immunostaining and Confocal Imaging

Whole mount immunostaining of the tibialis anterior (TA) muscle was performed as described (Vaden et al., 2015). Briefly, the TA muscle was immersed in ice-cold PBS containing 2% PFA for 1 h following dissection and immediately teased into thin bundles. Muscle bundles were then transferred to PBS containing 1% PFA and 1% Triton (PBS-T) and incubated overnight at 4°C with constant rocking. To improve visualization of axons and ultra-terminal sprouting, all mice used for NMJ immunostaining carried the *Thy1-YFP* transgene in addition to the transgene(s) of interest. Endplates were labeled by a 1 h room temperature incubation with rhodamine-conjugated α-bungarotoxin (α-BTX) and prepared for antibody application by a 1 h incubation in a blocking buffer containing 2% bovine serum albumin and 4% normal goat serum in PBS-T. For pJNK immunostaining, muscle bundles were incubated with primary antibody (pJNK, ##81E11, Cell Signaling Technology) for 5 days at 4°C with constant rocking. All images were captured using a Zeiss LSM 510 Meta confocal microscope (Carl Zeiss, Oberkochen, Germany). Endplate size was determined by tracing the circumference of the α-BTX-positive post-synaptic AChR cluster and computing area using ImageJ software (NIH, Bethesda, MD, USA).

### Statistical Analysis

All analyses were carried out using GraphPad Prism version for 6.0 for Mac OS X (GraphPad Software, La Jolla, CA, USA). For scaled variables, significant differences among genotypes were determined with a one-way ANOVA after assumptions for normality were verified with a D’Agostino and Pearson omnibus normality test with alpha set to 0.05. *Post hoc* comparisons were made with independent samples *t*-tests with Bonferroni-corrected alpha. In samples in which one or more genotype did not meet the assumption of normality, significant differences were determined with a Kruskal–Wallis test and *post hoc* comparisons were made with Mann–Whitney tests with Bonferroni-corrected alpha.

## Results

### Loss of USP14’s DUB Activity Increases Ubiquitin Conjugates

**FIGURE 1:**
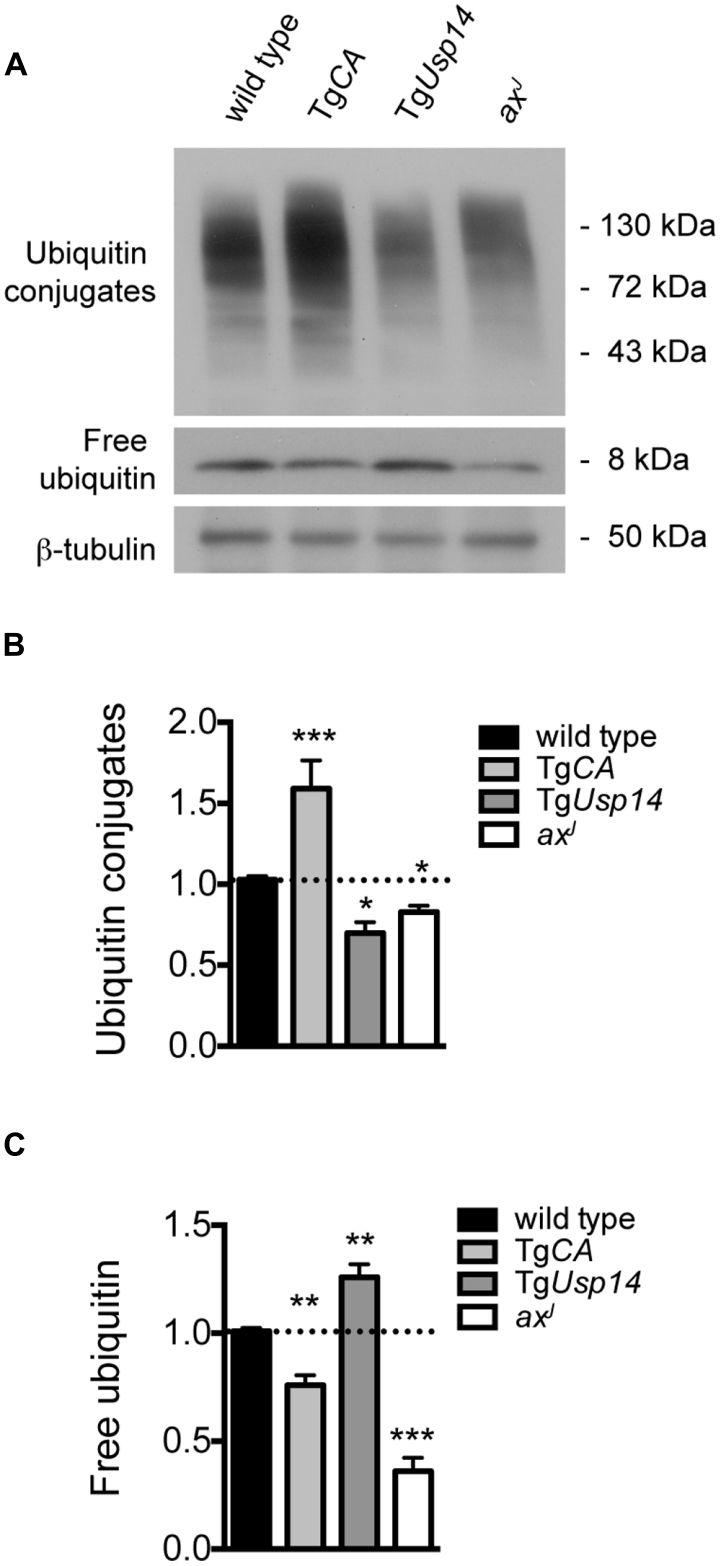
**Effects of manipulating USP14 expression on neuronal ubiquitin pools. (A)** Representative immunoblot of spinal cord lysates taken from 4-weeks-old wild type, Tg*CA*, Tg*Usp14*, and *ax^J^* mice, probed for ubiquitin. β-tubulin was used as a loading control. **(B)** Quantitation of the levels of ubiquitin conjugates, normalized to wild type levels; [*F*(3,30) = 20.35, *p* < 0.0001, one-way ANOVA]. **(C)** Quantitation of the levels of free ubiquitin, normalized to wild type levels; [*F*(3,30) = 59.15, *p* < 0.0001, one-way ANOVA]. Symbols represent unpaired *t*-tests compared against wild type and corrected for multiple comparisons with a Bonferroni adjustment, where ^∗^*p* < 0.05, ^∗∗^*p* < 0.01, ^∗∗∗^*p* < 0.001. *n* = at least three animals per genotype, run in duplicate.

To assess the interaction of USP14 with cellular ubiquitin pools, we performed immunoblot analysis on spinal cord extracts from wild type, Tg*CA*, and *ax^J^* mice (**Figure 1A**). Tg*Usp14* mice, which overexpress wild type USP14 in the nervous system (Crimmins et al., 2006), were included as a control. As previously reported (Anderson et al., 2005; Chen et al., 2009), loss of USP14 in the *ax^J^* mice resulted in significant decreases in free ubiquitin levels, whereas neuronal expression of catalytically inactive USP14 in the Tg*CA* mice led to only a modest reduction in free ubiquitin levels (**Figures 1A,C**). Instead, loss of USP14’s catalytic activity led to the retention of more proteins in a ubiquitinated state, as indicated by the increase in ubiquitin conjugates observed in the spinal cords of Tg*CA* mice compared to controls (**Figures 1A,B**). We also observed both an increase in free ubiquitin and a reduction in ubiquitin conjugates in the spinal cords of the mice overexpressing wild type USP14 (Tg*Usp14*), suggesting that USP14’s DUB activity makes a significant contribution to protein deubiquitination.

### Neuronal-Specific Over-Expression of Ubiquitin is Detrimental to Tg*CA* Mice

**FIGURE 2:**
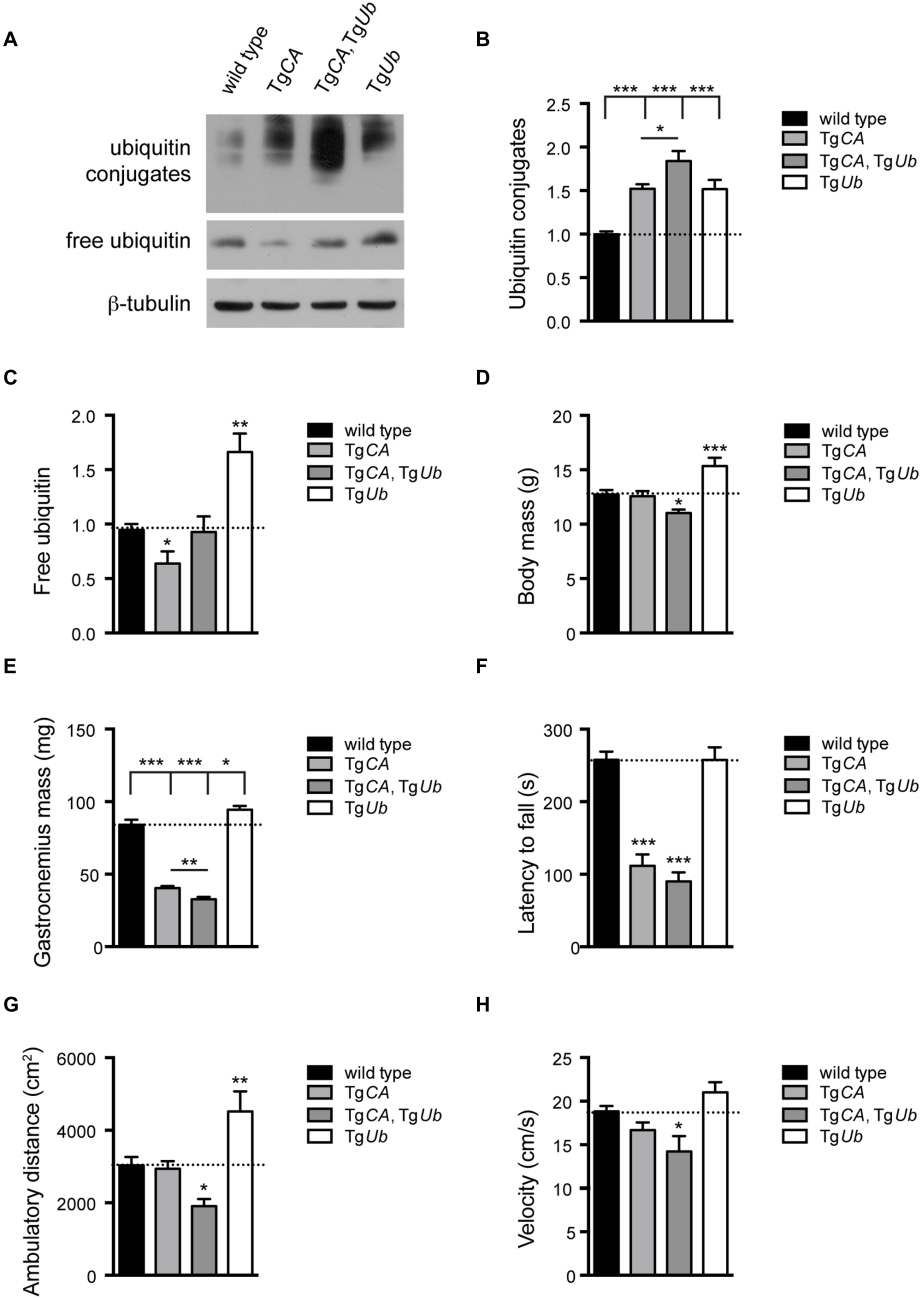
**Ubiquitin complementation does not rescue body mass, muscle mass, or motor deficits in Tg*CA* mice. (A)** Representative immunoblots of ubiquitin and USP14 from spinal cords of 4-weeks-old wild type; Tg*CA*, Tg*CA*, Tg*Ub*, and Tg*Ub* mice. β-tubulin was used as a loading control. **(B)** Quantitation of the levels of ubiquitin conjugates, normalized to wild type levels; [*F*(3,28) = 17.36, *p* < 0.0001, one-way ANOVA]. **(C)** Quantitation of the levels of free ubiquitin, normalized to wild type levels; [*F*(3,12) = 11.70, *p* < 0.001, one-way ANOVA]. **(D)** Body mass [*F*(3,56) = 9.03, *p* < 0.0001, one-way ANOVA] and **(E)** gastrocnemius muscle mass [*F*(3,70) = 94.72, *p* < 0.0001, one-way ANOVA] of 4-weeks-old mice. *n* = at least 12 animals per genotype. **(F)** Latency to fall from beam during a rotarod assay [*F*(3,26) = 38.36; *p* < 0.0001, one-way ANOVA]. **(G)** Total ambulatory distance [*F*(3,29) = 9.26; *p* < 0.001, one-way ANOVA] and **(H)** velocity [*F*(3,26) = 6.56; *p* < 0.01, one-way ANOVA] during 10 min open field assay. For **(F**–**H)**, *n* = at least five animals per genotype. All data are shown as mean ± SEM. Symbols represent unpaired *t*-tests compared against wild type and corrected for multiple comparisons with a Bonferroni adjustment, where ^∗^*p* < 0.05, ^∗∗^*p* < 0.01, ^∗∗∗^*p* < 0.001. In **(B,C)**, an additional unpaired *t*-test with Bonferroni adjustment was used to compare Tg*CA* and Tg*CA*,Tg*Ub* mice.

To test the hypothesis that increased ubiquitin conjugates, and not depletion of free ubiquitin, cause the motor neuron dysfunction in Tg*CA* mice, we restored free ubiquitin levels in the Tg*CA* mice by generating double transgenic mice expressing both Tg*Ub* and Tg*CA* under the neuronal *Thy1.2* promoter. The resulting Tg*CA*,Tg*Ub* mice expressed wild type levels of free ubiquitin as well as an increase in ubiquitin conjugates above what was observed in the Tg*CA* mice (Figures 2A–C). Consistent with our previous report (Vaden et al., 2015), there was no difference in body mass between 4-weeks-old Tg*CA* mice and controls (**Figure 2D**), but there was a significant reduction in the mass of the gastrocnemius muscles in the Tg*CA* mice compared to controls (**Figure 2E**). While transgenic expression of ubiquitin increased body and muscle mass in the *ax^J^* mice (Chen et al., 2011), both parameters were significantly reduced in the Tg*CA*,Tg*Ub* mice compared to both control and Tg*CA* mice (**Figures 2D,E**). Furthermore, whereas rotarod performance in *ax^J^*Tg*Ub* mice is improved over *ax^J^* mice (Chen et al., 2011), the Tg*CA*,Tg*Ub* mice did not perform better than Tg*CA* mice in this assay (**Figure 2F**). In fact, when we measured motor function in the less demanding open field assay, the Tg*CA*,Tg*Ub* mice showed reduced ambulatory distance (**Figure 2G**) and velocity (**Figure 2H**) compared to both wild type and Tg*CA* mice. In contrast, ubiquitin overexpression alone, in Tg*Ub* mice, caused increased body and gastrocnemius mass (**Figures 2D,E**) and ambulatory distance in the open field assay (**Figure 2G**) compared to controls.

**FIGURE 3:**
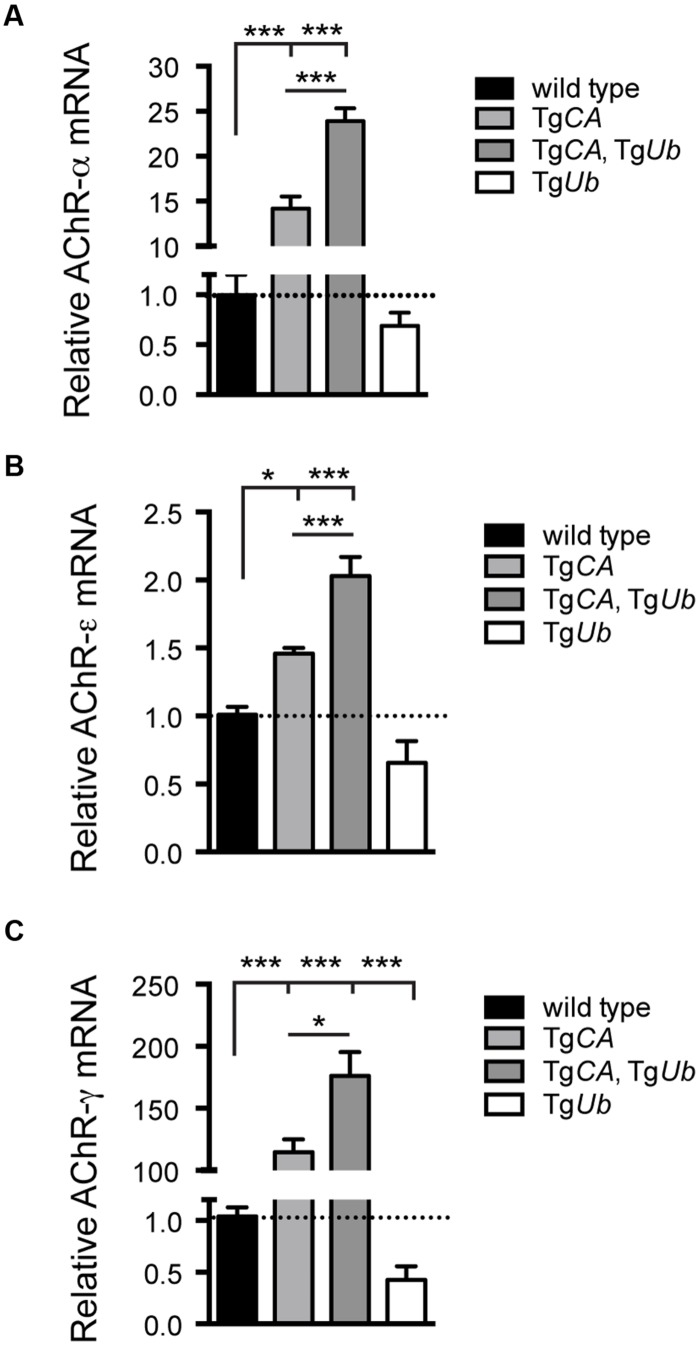
**Consequences of ubiquitin overexpression on AChR transcript levels in Tg*CA* mice.** qPCR analysis of *AChR* **(A)** –α [*F*(3,22) = 133.1; *p* < 0.0001, one-way ANOVA], **(B)** –ε [*F*(3,28) = 30.03; *p* < 0.0001, one-way ANOVA], and **(C)** –γ [*F*(3,19) = 35.56; *p* < 0.0001, one-way ANOVA] subunit mRNA abundance in 4-weeks-old wild type; Tg*CA*; Tg*CA*,Tg*Ub*, and Tg*Ub* mice expressed as relative fold change from wild type. *n* = at least four animals per genotype, run in triplicate. Data are shown as mean ± SEM. Symbols represent unpaired *t*-tests corrected for multiple comparisons with a Bonferroni adjustment where ^∗^*p* < 0.05, ^∗∗∗^*p* < 0.001.

We have previously shown that deficits in motor function and NMJ synaptic transmission are inversely related to muscle *AChR* transcript levels, and, in particular, that expression of the fetal *AChR-*γ subunit is dramatically increased in adult animals with motor and synaptic deficits (Chen et al., 2009, 2011; Vaden et al., 2015). As predicted by their poor open field performance, *AChR-*α, -ε, and *-*γ transcripts were significantly increased in Tg*CA*,Tg*Ub* mice over all other genotypes, including Tg*CA* (**Figures 3A–C**). Finally, there was a significant decrease in *AChR-*γ transcripts in Tg*Ub* mice compared to controls (**Figure 3C**), and a trend toward decreased *AChR-*α and *-*ε transcripts (**Figures 3A,B**). Together with our previous report, these data demonstrate that increased ubiquitin expression in the presence of catalytically inactive USP14 (in the Tg*CA*,Tg*Ub* mice) has drastically different effects than when USP14 is absent (*ax^J^*Tg*Ub* mice).

### Ubiquitin has a Dose-Dependent Effect on Motor Function in Wild Type Mice

**FIGURE 4:**
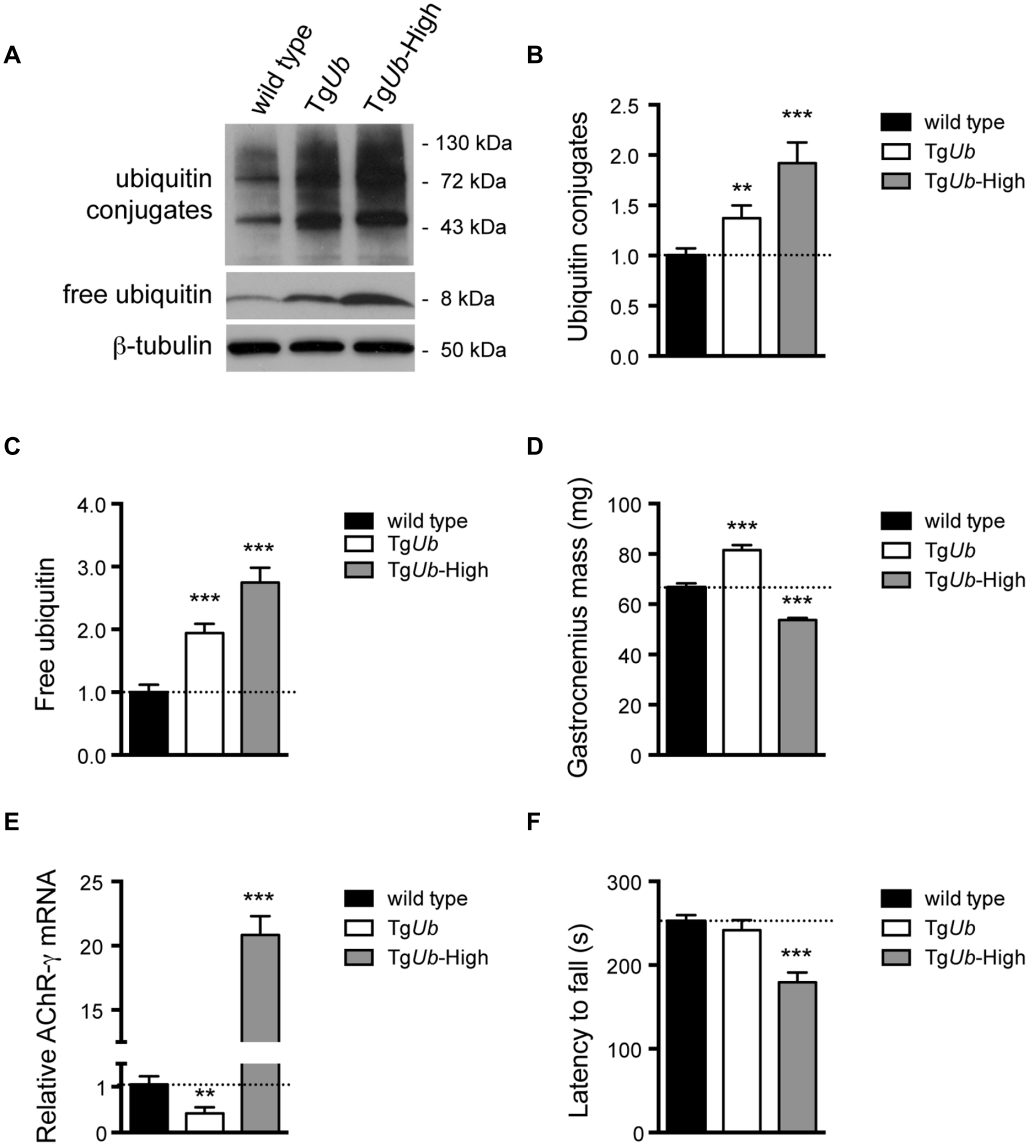
**Dose-dependent effects of ubiquitin on muscle development and motor function. (A)** Representative immunoblots of free and conjugated ubiquitin in spinal cords from two lines of transgenic mice over-expressing ubiquitin under the neuronal *Thy1.2* promoter. β-tubulin was used as a loading control. **(B)** Quantitation of the levels of ubiquitin conjugates, normalized to wild type levels; [*F*(2,39) = 10.10, *p* < 0.001, one-way ANOVA]. **(C)** Quantitation of the levels of free ubiquitin, normalized to wild type levels; [*F*(2,38) = 24.01, *p* < 0.0001, one-way ANOVA]. **(D)** Comparison of gastrocnemius muscle mass from 4- to 5-weeks-old female mice; [*F*(2,23) = 89.83, *p* < 0.0001, one-way ANOVA]. The same effect was observed in male mice; [*F*(2,19) = 17.35, *p* < 0.0001, one-way ANOVA]. **(E)** Comparison of *AChR*-γ transcript abundance, normalized to wild type [*F*(2,12) = 185.5, *p* < 0.0001]. For **(B**–**E)** symbols represent unpaired *t*-tests compared against wild type and corrected for multiple comparisons with a Bonferroni adjustment where ^∗∗^*p* < 0.01, ^∗∗∗^*p* < 0.001. **(F)** Latency to fall from beam during a rotarod assay (*H* = 12.22, 1 df, *p* < 0.01, Kruskal–Wallis test), symbol represents Mann–Whitney test compared against wild type and corrected for multiple comparisons with a Bonferroni adjustment where ^∗∗∗^*p* < 0.001. For **(D**–**F)**, *n* = at least five animals per genotype. All data are shown as mean ± SEM.

Loss of USP14’s catalytic activity leads to a greater increase in ubiquitin conjugates in Tg*CA*,Tg*Ub* spinal cords than ubiquitin overexpression alone causes in the spinal cords of Tg*Ub* mice (**Figure 2**). To determine whether increased protein ubiquitination in the nervous system could alter neuromuscular function independently of reduced USP14 activity, we compared muscle mass, *AChR* expression, and rotarod performance in the Tg*Ub* mice used in previous experiments with another transgenic founder line (Tg*Ub-*H) that has higher levels of ubiquitin expression (**Figures 4A–C**). The gastrocnemius muscles of 4- to 6-weeks-old female Tg*Ub* mice were significantly larger than those of controls (**Figure 4D**). In contrast, the increased ubiquitin expression in the Tg*Ub-H* mice resulted in significantly decreased gastrocnemius mass compared to controls (**Figure 4D**). The same was true for male wild type, Tg*Ub*, and Tg*Ub-*H mice (data not shown). Further, we found that Tg*Ub-*H mice had a significant increase in the abundance of fetal *AChR-*γ transcripts (**Figure 4E**), a marker of synaptic deficits and motor dysfunction in adult *ax^J^* animals (Chen et al., 2009, 2011). Finally, whereas overexpression of ubiquitin in the Tg*Ub* mice had no effect on rotarod performance, increased levels of ubiquitin in the Tg*Ub-*H mice caused them to fall from the rotating beam more quickly than controls (**Figure 4F**), and display an abnormal gait while performing this task (data not shown). Together, these data show that ubiquitin has dose-dependent effects on motor function and muscle development.

### Ubiquitin Overexpression Corrects Presynaptic Structural Deficits at the Tg*CA* NMJ

**FIGURE 5:**
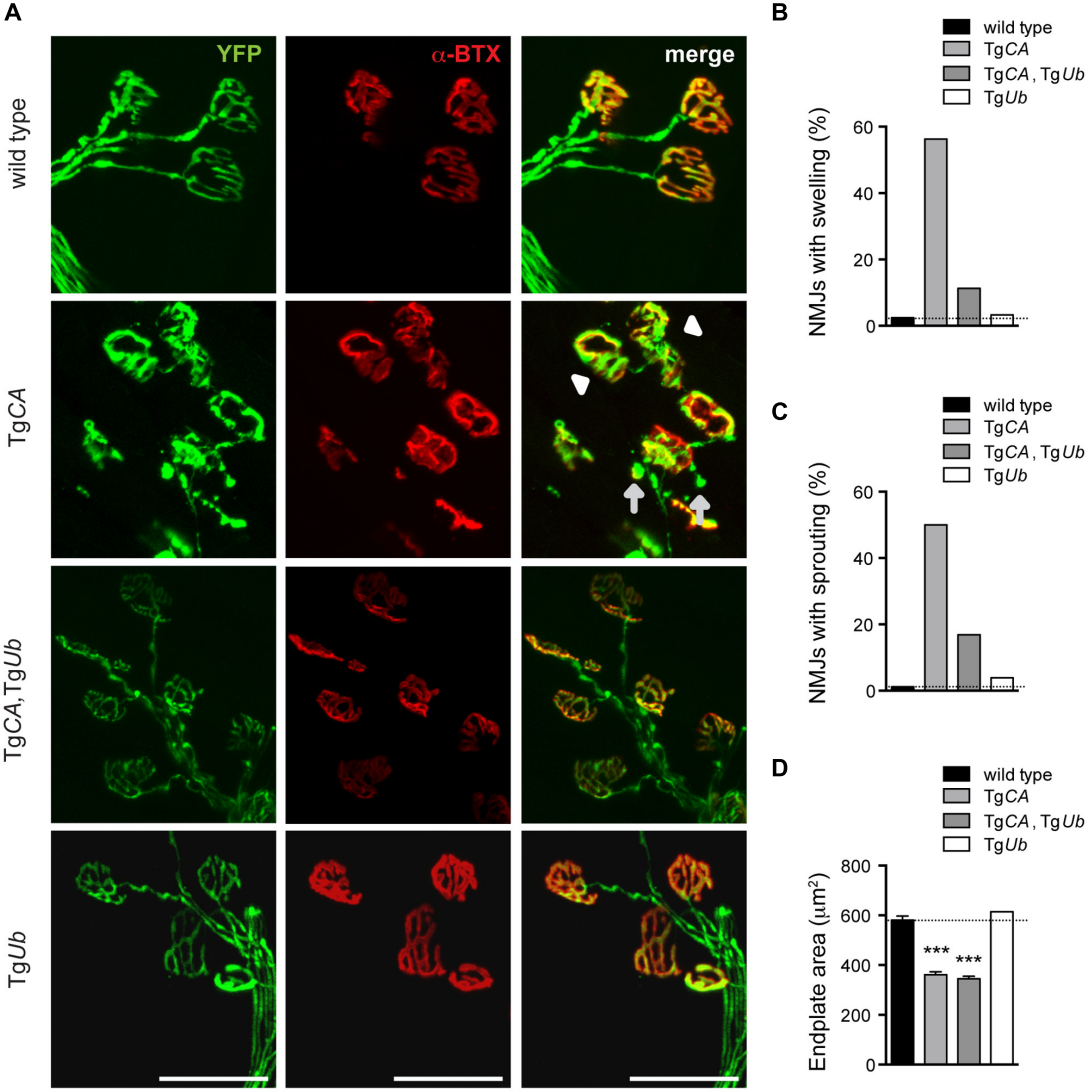
**Ubiquitin complementation improves NMJ structure in Tg*CA* mice. (A)** Whole-mount immunostaining of tibialis anterior (TA) muscles from 4- to 5-weeks-old wild type; Tg*CA*, Tg*CA*, Tg*Ub*, and Tg*Ub* mice expressing YFP (green) under the neuronal *Thy1.2* promoter. AChR clusters were visualized with rhodamine-conjugated α-bungarotoxin (α-BTX, red). Arrowhead indicates terminal swelling and arrows indicate ultra-terminal sprouting. Scale bar represents 50 μM. **(B)** Percent of NMJs with presynaptic swellings or **(C)** sproutings. **(D)** Quantitation of α-BTX-positive endplate area shown as mean ± SEM. [*F*(3,564) = 104.60; *p* < 0.0001, one-way ANOVA]. Symbols in **(C)** represent unpaired *t*-tests compared against wild type and corrected for multiple comparisons with a Bonferroni adjustment, where ^∗∗∗^*p* < 0.001. For **(B**–**D)**, *n* = at least 130 NMJs from five animals.

We have previously reported that the NMJs of 4- to 6-weeks-old USP14-deficient *ax^J^* mice have poor motor endplate arborization, swollen presynaptic terminals, and ultra-terminal sprouting, and that these deficits are corrected in *ax^J^*Tg*Ub* mice (Chen et al., 2011). Because Tg*CA* mice recapitulate these *ax^J^* NMJ deficits (Vaden et al., 2015), we investigated whether NMJ structure was improved in Tg*CA*,Tg*Ub* mice (**Figure 5A**). Whereas 56% of Tg*CA* NMJs had presynaptic swellings, ubiquitin overexpression improved NMJ structure and only 11% of Tg*CA*,Tg*Ub* terminals were swollen (**Figure 5B**). Similarly, we observed ultra-terminal or ultra-axonal sprouting in 50% of the terminals of the Tg*CA* mice compared to only 17% in the Tg*CA*,Tg*Ub* mice (**Figure 5C**). Terminal swelling and sprouting were seen only rarely in wild type and Tg*Ub* mice (**Figures 5B,C**). We found a significant effect of genotype on endplate area, with smaller, more plaque-like endplates in the Tg*CA* and Tg*CA*,Tg*Ub* mice than wild type and Tg*Ub* mice (**Figure 5D**), consistent with the increase in *AChR* mRNA abundance observed in these mice (**Figure 3**).

### Ubiquitin Complementation Reduces pJNK at the NMJs, but Not in the Spinal Cords, of the Tg*CA* Mice

**FIGURE 6:**
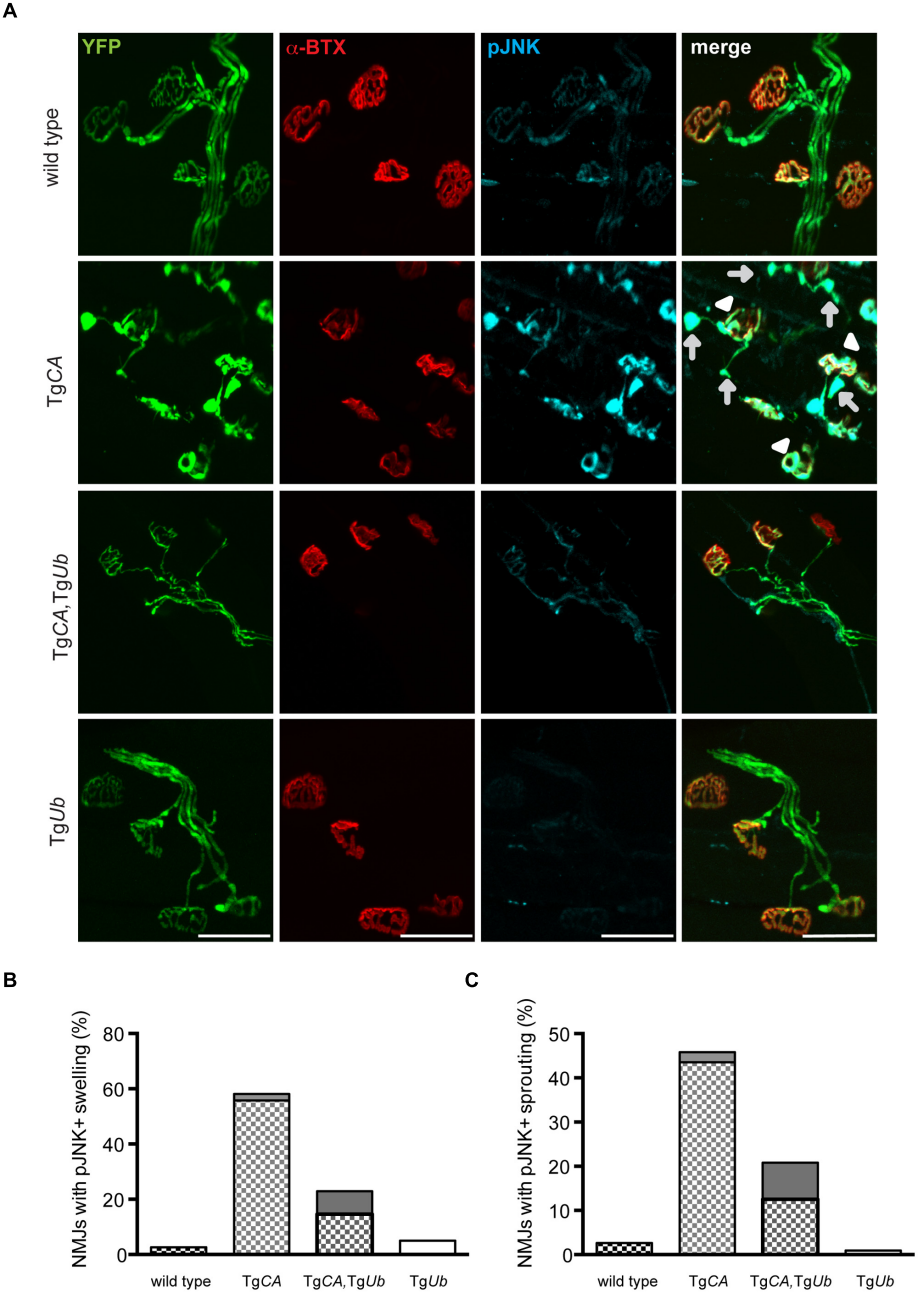
**Ubiquitin complementation reduces pJNK levels at the NMJs of Tg*CA* mice. (A)** Whole-mount immunostaining of TA muscles from 4- to 5-weeks-old wild type; Tg*CA,* Tg*CA*, Tg*Ub*, and Tg*Ub* mice expressing YFP (green) under the neuronal *Thy1.2* promoter using an antibody against pJNK (light blue). Endplates were visualized with rhodamine-conjugated α-bungarotoxin (α-BTX, red). Arrowheads indicate pJNK-positive terminal swelling and arrows indicate pJNK-positive ultra-terminal sprouting. Scale bar represents 50 μM. Quantitation of **(B)** presynaptic swellings or **(C)** ultra-terminal sproutings. The patterned portion of each bar represents pJNK-positive swellings and the height of the bar represents the total percent of swollen NMJs. *n* = at least 60 NMJs from three animals per genotype.

**FIGURE 7:**
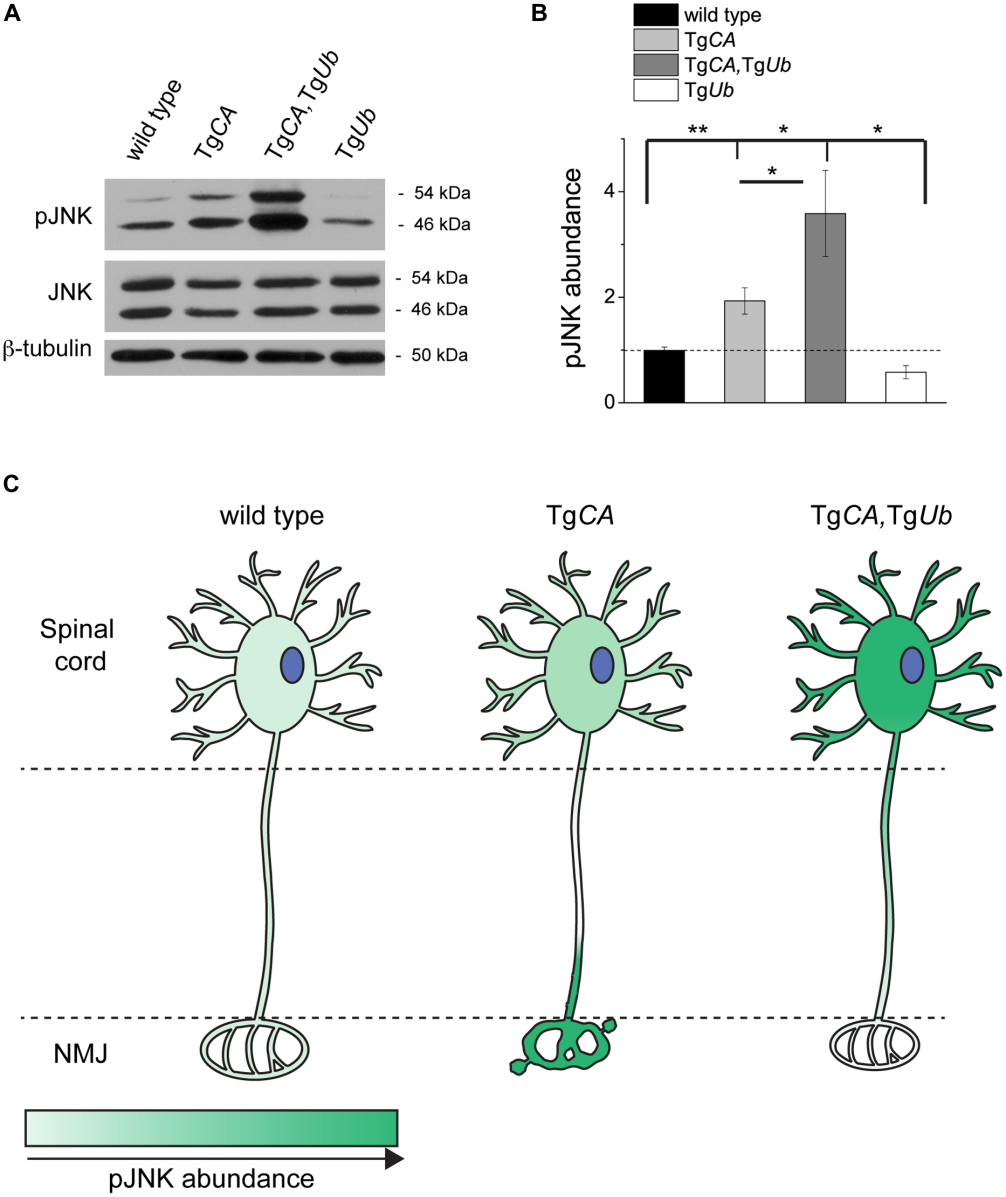
**Ubiquitin overexpression does not reduce pJNK abundance in Tg*CA* spinal cords. (A)** Representative immunoblots of pJNK and total Jun N-terminal kinase (JNK) from spinal cords of 4 weeks-old wild type, Tg*CA*, Tg*CA*,Tg*Ub*, and Tg*Ub* mice. β-tubulin was used as a loading control. **(B)** Quantitation of pJNK levels in 4-weeks-old mice normalized to wt and total JNK abundance [*F*(3,36) = 9.532; *p* < 0.0001, one-way ANOVA]. Symbols represent unpaired *t*-tests corrected for multiple comparisons with a Bonferroni adjustment, where ^∗^*p* < 0.05, ^∗∗^*p* < 0.01. **(C)** Schematic representation of the levels of pJNK abundance observed in the spinal cords, distal axons of motor neurons, and NMJs of 4- to 5-weeks-old mice of the genotypes indicated, where darker green indicates greater pJNK abundance.

We recently reported that pJNK is a prominent component of the terminal swellings and sproutings in Tg*CA* mice, and that the extent of this pathology is reduced by treatment with the JNK inhibitor SP600125 (Vaden et al., 2015). Because ubiquitin complementation improved the structure of the Tg*CA* endplates (**Figure 5**), we investigated whether this reduction in pathology was associated with a reduction of pJNK at the NMJ. As previously reported, we observed strong pJNK immunostaining in the presynaptic terminals of Tg*CA* mice (**Figure 6A**): 55.8% of terminals contained pJNK-positive swellings (**Figure 6B**) and 43.5% of terminals contained pJNK-positive ultra-terminal sprouts (**Figure 6C**). In contrast, ubiquitin overexpression in the Tg*CA*,Tg*Ub* mice reduced the pathology, and only 8.3% of terminals contained pJNK-positive swelling or sprouting (**Figures 6B,C**). pJNK-positive pathology was negligible in wild type mice and absent in Tg*Ub* mice. However, when we immunoblotted spinal cord lysates from wild type, Tg*CA*, Tg*CA*,Tg*Ub*, and Tg*Ub* mice with a pJNK-specific antibody, we found that the levels of pJNK in Tg*CA*,Tg*Ub* mice were elevated over the levels observed in Tg*CA* mice, and that spinal cord lysates of both genotypes had significantly more pJNK than spinal cord lysates from wild type mice (**Figures 7A,B**). There was no change in total JNK abundance in the Tg*CA* or Tg*CA*,Tg*Ub* mice compared to controls, indicating that USP14 and ubiquitin regulate JNK activation and not JNK stability. We also observed a significant decrease in the abundance of pJNK, but not total JNK, in Tg*Ub* spinal cord lysates compared to controls (**Figures 7A,B**). A schematic summarizing the levels of pJNK abundance observed in the spinal cords, distal motor neuron axons, and NMJs of wild type, Tg*CA*, and Tg*CA*,Tg*Ub* mice is shown in **Figure 7C**.

## Discussion

Three main conclusions can be drawn from these studies. First, overexpression of ubiquitin increased the abundance of ubiquitin conjugates in the spinal cord (**Figure 4**), demonstrating that free ubiquitin is a limiting factor in some ubiquitin-dependent processes in the nervous system. Second, USP14’s ubiquitin hydrolase activity plays a significant role in regulating the distribution of free and conjugated ubiquitin pools in neurons (**Figure 1**). The increased abundance of ubiquitin conjugates, and not depletion of the free ubiquitin pool, directly correlated with impaired motor function when USP14’s ubiquitin hydrolase activity was inhibited (**Figures 2** and **3**). Third, both ubiquitin levels and the catalytic activity of USP14 impact the abundance of pJNK at the NMJ and in the spinal cord (**Figures 6** and **7**) and, in turn, the levels of pJNK at the NMJ impact the structure of the presynaptic terminal.

Neuronal overexpression of ubiquitin led to increased ubiquitin conjugates in a dose-dependent manner in two transgenic founder lines, Tg*Ub* and Tg*Ub-H*, with low and high levels of ubiquitin overexpression, respectively (**Figure 4A**). These ubiquitin conjugates had dose-dependent effects on motor function and muscle development in wild type mice (**Figure 4**). Mild ubiquitin overexpression led to increased muscle development and reduced expression of the fetal γ subunit of the muscle *AChR*, which is correlated with enhanced NMJ synaptic transmission (Chen et al., 2009, 2011; Vaden et al., 2015), in Tg*Ub* mice compared to controls (**Figures 4B–D**). In contrast, more dramatic ubiquitin overexpression reduced muscle development, increased *AChR-*γ abundance, and hindered motor performance in the Tg*Ub-*H mice compared to controls (**Figures 4B–D**). Moreover, we have previously reported that the Tg*Ub*-H mice develop adult-onset motor endplate disease (Hallengren et al., 2013). Together, these data highlight the importance of maintaining ubiquitin levels within a relatively narrow window.

Ubiquitin-specific protease 14 is an important regulator of ubiquitin homeostasis. We found that increasing the level of wild type USP14 in the nervous system resulted in an increase in the free ubiquitin pool and decreased levels of ubiquitin conjugates as compared to controls. Because both the *ax^J^* mice and Tg*CA* mice exhibit severe motor endplate disease, we were surprised to find differences in ubiquitin homeostasis between these two mouse lines. Loss of USP14 in the *ax^J^* mice significantly reduced the abundance of both free and conjugated ubiquitin. In contrast, expression of catalytically inactive USP14CA in the Tg*CA* mice led to a 40% increase in ubiquitin conjugates and a 25% reduction of free ubiquitin. Further studies will be required to determine if these effects of USP14 on ubiquitin pools are due to changes in ubiquitin-dependent degradation by the proteasome. These differences in ubiquitin homeostasis in the spinal cords of the *ax^J^* and Tg*CA* mice, along with the dose-dependent impact of increased ubiquitin conjugates on motor function and muscle development, may provide a framework for understanding the opposite effects of ubiquitin overexpression in the *ax^J^* and Tg*CA* mice. One possibility for these differences is that USP14CA may have an increased affinity for its substrates compared to USP14. Binding of USP14CA may slow their eventual deubiquitination by another DUB and result in prolonged ubiquitin-dependent signaling. Alternatively, increased affinity of USP14CA to ubiquitin conjugates may block their proteasomal degradation and lead to protein aggregation that could affect synaptic function.

In the *ax^J^*, Tg*Ub* mice, the levels of ubiquitin conjugates and free ubiquitin are slightly elevated over what is normally observed in wild type mice (Chen et al., 2011). We have now shown that a modest increase in ubiquitin conjugates and free ubiquitin in the Tg*Ub* mice correlated with increased muscle development and motor function, even when wild type USP14 was present (**Figure 4**). In contrast, the levels of ubiquitin conjugates in the spinal cords of the Tg*CA*, Tg*Ub* mice were nearly twofold what is observed in wild type mice (**Figures 2A,B**), and reduced muscle development and mobility were observed in the Tg*CA*,Tg*Ub* mice compared to both wild type and Tg*CA* mice. Our studies of the Tg*Ub-*H mice indicated that a twofold increase in ubiquitin conjugates resulted in deficits in motor function and muscle development even when USP14’s catalytic activity was intact (**Figure 4**). Together, these data open the possibility that the restoration of motor neuron function in *ax^J^*Tg*Ub* mice is indirect and can be attributed to increased ubiquitin conjugates, while suggesting that ubiquitin overexpression aggravates the accumulation of ubiquitin conjugates caused by loss of USP14’s DUB activity in Tg*CA* mice to exaggerate the existing phenotype (**Figures 2** and **3**). Since both increases and decreases in ubiquitin pools are associated with deleterious effects in cells and animals models (Parag et al., 1987; Ferguson et al., 1990; Osaka et al., 2003; Anderson et al., 2005; Hirsch et al., 2006) the ubiquitin depletion observed in the absence of functional USP14 may contribute to other neurological deficits observed in the Tg*CA* and *ax^J^* mice.

However, when considered together with our previous work demonstrating a role for pJNK in the NMJ pathology observed in the Tg*CA* mice (Vaden et al., 2015), this study suggests that ubiquitin complementation corrects these structural deficits directly, by reducing pJNK-positive pathology (**Figures 5** and **6**). While we have not examined pJNK abundance at the NMJs of *ax^J^* mice, the increased JNK activation in *ax^J^* spinal cords suggests that the mechanism underlying NMJ pathology caused by loss of USP14 is the same as that caused by loss of USP14’s DUB activity (Vaden et al., 2015). Given the reduction of pJNK at Tg*CA,*Tg*Ub* NMJs compared to Tg*CA* NMJs (**Figure 6**), we were surprised by the dramatic increase in pJNK levels in the spinal cords of the Tg*CA*,Tg*Ub* mice (**Figures 7A,B**). However, we have recently reported that loss of USP14’s DUB activity leads to enhanced K63-linked ubiquitination of MLK3 (Vaden et al., 2015), which drives it to dimerize, autophosphorylate, and activate its immediate downstream targets, MKK4/7, which, in turn, activate JNK (Humphrey et al., 2013). It is therefore possible that the increased JNK activation that we observed in Tg*CA*,Tg*Ub* spinal cords compared to Tg*CA* spinal cords (**Figures 7A,B**) results from increased activation of MLK3.

The means by which ubiquitin complementation reduced pJNK-positive pathology at the NMJ remains unclear. One possibility is that the increased ubiquitin in Tg*CA*,Tg*Ub* mice stimulates the degradation of pJNK or its upstream kinases at the NMJ or, alternatively, that ubiquitin contributes to the activation of phosphatases that act on pJNK. Both of these explanations are consistent with the reduction of pJNK abundance in Tg*Ub* spinal cords compared to wild type, but not with the increase in pJNK levels over both wild type and Tg*CA* levels in the spinal cords of the Tg*CA*,Tg*Ub* mice (**Figures 7A,B**). In contrast, the well-documented retrograde axonal transport of activated JNK (Cavalli et al., 2005; Lindwall and Kanje, 2005; Shin et al., 2012; Drerup and Nechiporuk, 2013) and the pJNK-positive swellings at Tg*CA* NMJs (**Figure 6**) are all consistent with a deficit in the retrograde transport of pJNK out of axon terminals in the Tg*CA* mice. The lack of pJNK-positive pathology in the nerve terminals (**Figure 6**) of the Tg*CA*,Tg*Ub* mice, combined with the robust elevation of pJNK abundance in Tg*CA*,Tg*Ub* spinal cords (**Figures 7A,B**), the site of motor neuron cell bodies, suggest that ubiquitin may stimulate the retrograde axonal transport of pJNK (**Figure 7C**). Although a role for ubiquitin in retrograde axonal transport has not been demonstrated directly, it was recently reported that altered ubiquitin homeostasis contributes to the deficits in TrkB retrograde transport caused by the exposure of cultured neurons to Aβ oligomers (Poon et al., 2013).

Finally, our findings also suggest that the functional deficits observed in the Tg*CA* and *ax^J^* mice do not arise because of altered NMJ structure. This is consistent with our previous report that acute inhibition of USP14 at the NMJs of wild type mice causes deficits in synaptic transmission that are similar to what is observed in the Tg*CA* and *ax^J^* mice, while intramuscular injections of the same inhibitor given over the course of a week do not cause NMJ pathology (Vaden et al., 2015). Together, these findings may indicate that USP14 regulates synapse structure and function through distinct pathways. The same is true of the ubiquitin ligase highwire, which regulates the structure and function of the *Drosophila* NMJ through separate pathways (Collins et al., 2006).

## Conflict of Interest Statement

Scott Wilson is a paid consultant for Progenera Inc. The collection, analysis, and interpretation of the data presented in this manuscript were not influenced by my relationship with Progenra Inc.

## Abbreviations

Tg*CA*: (transgene expressing catalytically inactive USP14, generated by mutating the active site cysteine to an alanine residue, expressed under the neuronal *Thy1.2* promoter; also refers to mice expressing this transgene)
Tg*Ub*: (transgene expressing ubiquitin under the *Thy1.2* promoter; also refers to mice expressing this transgene)
Tg*Ub-H*: (a transgenic founder line with more robust ubiquitin overexpression than Tg*Ub*)
Tg*CA*: Tg*Ub* (mice expressing both Tg*CA* and Tg*Ub*).
